# Soluble TNF Mediates the Transition from Pulmonary Inflammation to Fibrosis

**DOI:** 10.1371/journal.pone.0000108

**Published:** 2006-12-27

**Authors:** Nikos Oikonomou, Vaggelis Harokopos, Jonathan Zalevsky, Christos Valavanis, Anastasia Kotanidou, David E. Szymkowski, George Kollias, Vassilis Aidinis

**Affiliations:** 1 Institute of Immunology, Biomedical Sciences Research Center “Alexander Fleming,” Athens, Greece; 2 Xencor, Inc., Monrovia, California, United States of America; 3 Molecular Pathology Unit, Department of Pathology, Metaxa Cancer Hospital, Piraeus, Greece; 4 First Department of Critical Care, Medical School, University of Athens, Athens, Greece; Rockefeller University, United States of America

## Abstract

**Background:**

Fibrosis, the replacement of functional tissue with excessive fibrous tissue, can occur in all the main tissues and organ systems, resulting in various pathological disorders. Idiopathic Pulmonary Fibrosis is a prototype fibrotic disease involving abnormal wound healing in response to multiple sites of ongoing alveolar epithelial injury.

**Methodology/Principal Findings:**

To decipher the role of TNF and TNF-mediated inflammation in the development of fibrosis, we have utilized the bleomycin-induced animal model of Pulmonary Fibrosis and a series of genetically modified mice lacking components of TNF signaling. Transmembrane TNF expression is shown to be sufficient to elicit an inflammatory response, but inadequate for the transition to the fibrotic phase of the disease. Soluble TNF expression is shown to be crucial for lymphocyte recruitment, a prerequisite for TGF-b1 expression and the development of fibrotic lesions. Moreover, through a series of bone marrow transfers, the necessary TNF expression is shown to originate from the non-hematopoietic compartment further localized in apoptosing epithelial cells.

**Conclusions:**

These results suggest a primary detrimental role of soluble TNF in the pathologic cascade, separating it from the beneficial role of transmembrane TNF, and indicate the importance of assessing the efficacy of soluble TNF antagonists in the treatment of Idiopathic Pulmonary Fibrosis.

## Introduction

Fibroproliferative diseases are among the leading causes of morbidity and mortality worldwide. Fibrosis, the replacement of functional tissue with excessive fibrous tissue, can occur in all the main tissues and organ systems resulting in various disorders including cardiac, cerebral and peripheral vascular disease, liver cirrhosis, progressive kidney disease and a number of interstitial lung diseases such as idiopathic pulmonary fibrosis (IPF). Despite their obvious etiological and clinical distinctions, most of these fibrotic diseases have in common a persistent inflammatory stimulus that sustains and/or stimulates the production of growth factors and fibrogenic cytokines, which together stimulate the deposition of connective-tissue elements that progressively remodel and destroy normal tissue architecture.

IPF (also known as cryptogenic fibrosing alveolitis) is a chronic, interstitial, fibrotic lung disease, with a prevalence of 7–10∶100000 (increasing with age) and a mean survival of 3–4 years (decreasing with age). Clinically, IPF is characterized by progressive, exertional dyspnea and nonproductive cough, worsening of pulmonary function and radiographically evident interstitial infiltrates (honeycombing). Histologically, IPF is associated with the appearance of Usual Interstitial Pneumonitis (UIP), which is characterized by patchy subpleural and/or paraseptal interstitial fibrosis alternating with areas of mild inflammation and normal lung. The hallmark of IPF/UIP is the distinctive presence of fibroblastic foci and exuberant Extracellular Matrix (ECM) deposition, leading to thickening of alveolar septa and the collapse of normal lung architecture. Due to the lack of a more effective alternative, the fundamental therapeutic approach has been use of corticosteroids, alone or in combination with other immunosuppressive agents; however, this has little impact on long-term survival [1], [2].

Although the etiology and pathogenesis of IPF remain poorly understood, a number of conditions and risk factors are weakly associated with the disease: cigarette smoking, occupational/environmental factors, latent viral infections, as well as age/gender/genetic predisposition. The occurrence of pulmonary fibrosis (with a UIP histological pattern) as a side effect in humans receiving bleomycin (BLM) for cancer chemotherapy led to the development of a BLM-induced animal model of pulmonary fibrosis (BLM/PF). Despite the intrinsic limitations and the evolutionary distance, the BLM/PF animal model shares many clinical features with the human disease, and therefore provides valuable insights into the pathogenetic mechanisms that govern disease activation and perpetuation. Utilization of this model, as well as site-specific and/or temporal overexpression or ablation of candidate pathogenic genes, is responsible for most of our knowledge concerning IPF pathogenesis. In this context, current research suggests that the mechanisms driving IPF reflect abnormal, deregulated wound healing within the lung, involving increased activity and possibly exaggerated responses by a spectrum of proinflammatory and profibrogenic factors [1]–[7].

Tumor Necrosis Factor (TNF) is a pleiotropic cytokine expressed by many cell types in response to infection or injury. TNF affects multiple responses that extend well beyond its well-characterized pro-inflammatory properties to include diverse signals for cellular differentiation, proliferation and death; its functions can be both beneficial, as well as detrimental. Inappropriate production of TNF has been implicated in the pathogenesis of a variety of human diseases, including sepsis, cerebral malaria, diabetes, cancer, osteoporosis, allograft rejection, multiple sclerosis, rheumatoid arthritis, and inflammatory bowel diseases [8], [9]. Increased levels of TNF have been found in IPF patients [10]–[13], as well as in various animal models of PF [14]–[18], while TNF polymorphisms have been associated with an increased risk of developing the disease [19].

In order to decipher the role of TNF and TNF-mediated inflammation in the development of PF, we have systematically administered intravenous BLM to genetically modified mice of the same susceptible genetic background, lacking components of TNF signaling in the hematopoietic and/or the non-hematopoietic cellular compartments. Transmembrane (tm) TNF expression from non-hematopoietic cells was shown to be sufficient to elicit an inflammatory response to BLM, while soluble (sol) TNF to be necessary for appropriate lymphocyte recruitment, TGF-b1 expression and the development of fibrotic lesions.

## Results

### TNF is necessary for the development of PF

**Figure 1:**
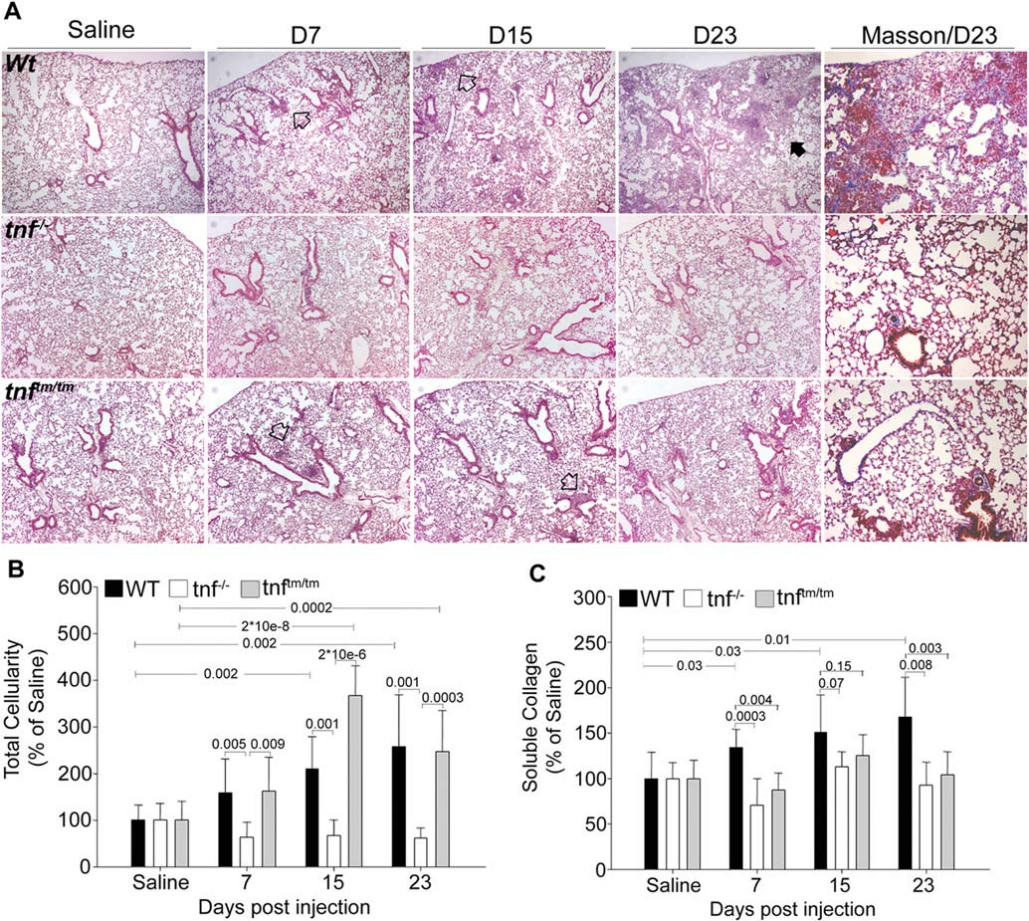
solTNF is necessary for BLM-induced PF WT and transgenic mice were injected intravenously with saline or BLM and sacrificed at indicated time points post injection. (A) Representative H/E (4×) and Masson (10×) stainings of lung sections of WT, *tnf^−/−^* and *tnf^tm/tm^* mice. Inflammatory infiltrates are evident in WT and *tnf^tm/tm^* lungs at subpleural and peribronchial areas (open arrow) while fibrosis develops only in WT lungs (closed arrow). *tnf^−/−^* mice show no signs of disease. (B) Total inflammatory cell counts in BALF, expressed as a percentage over the corresponding saline injections. (C) Soluble collagen determination in lung extracts, expressed as a percentage of the corresponding saline injections. Bars represent mean values ±SD. Statistically significant differences are indicated by the corresponding t-test p values.

To decipher the role of TNF in the development of PF we have administered BLM to genetically modified mice (of the same susceptible genetic background; C57/Bl6) lacking components of TNF signaling. In order to compare multiple mouse lines, experimental procedures were standardized as described in detail in Figure S1. As expected, BLM administration in WT mice resulted in progressive subpleural/peribronchial pulmonary inflammation, which subsequently diffused into the parenchyma (Fig. 1A; WT). Accordingly, alveolar inflammatory cells, as measured in bronchoalveolar lavage fluid (BALF), increased gradually to peak 23 d post BLM injection (Fig. 1B; WT). Inflammation was followed by the development of mainly subpleural and peribronchial fibrotic patches, characterized by alveolar septa thickening and focal dilation of respiratory bronchioles and alveolar ducts (Fig. 1A; WT). Concomitantly, collagen accumulation peaked 23 d post BLM injection, as measured in lung protein extracts with a soluble collagen assay (Fig. 1C; WT). In sharp contrast, BLM-injected *tnf^−/−^* mice were completely protected from the development of disease, exhibiting no inflammation, lack of collagen accumulation and normal lung architecture (Fig. 1A,B,C; *tnf^−/−^*). Thus, TNF expression is necessary for collagen accumulation and the development of BLM-induced PF.

### Inflammatory responses elicited by transmembrane TNF do not support the development of PF

**Figure 2:**
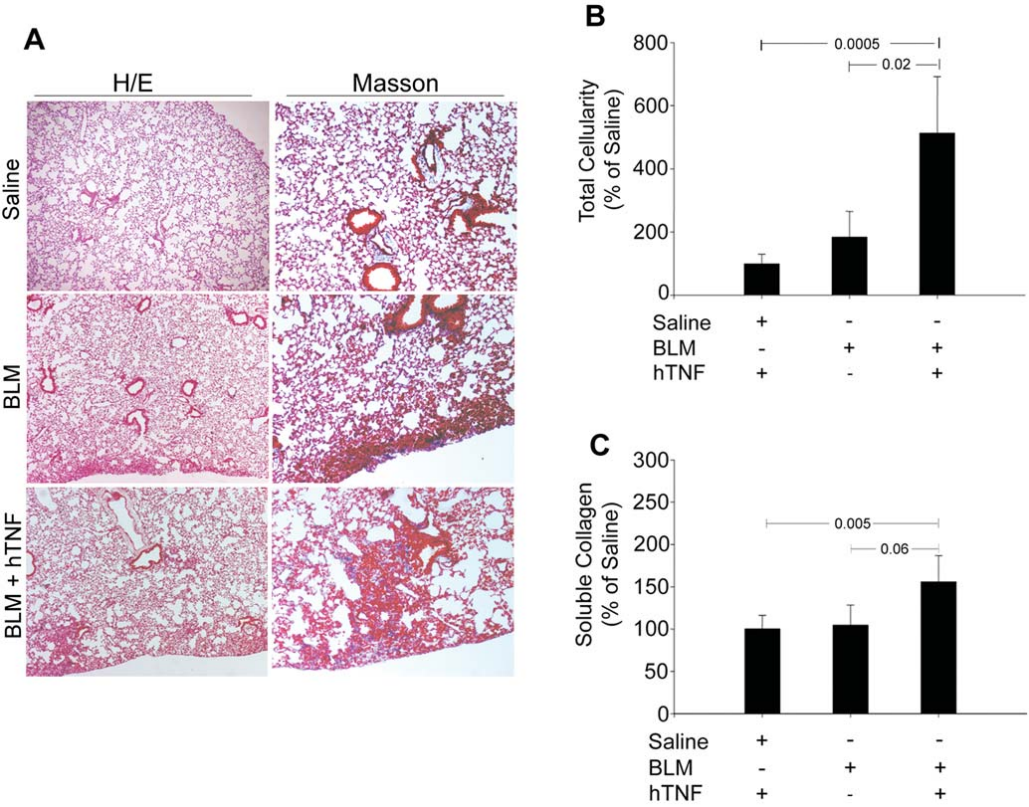
solTNF administration restores disease potential in *tnf^tm/tm^* mice *tnf^tm/tm^* mice received either aerosolized recombinant hTNF (200 ng/ml) or saline prior to injection with BLM. 23 days post injection, mice were sacrificed for pathology evaluation. Parallel treatment with solTNF and BLM resulted in the development of fibrotic areas in the lungs of *tnf^tm/tm^* mice. (A) Representative H/E (4×) and Masson (10×) stainings of lung sections. (B) Total inflammatory cell counts in BALF, expressed as a percentage over the corresponding saline injections. (C) Soluble collagen determination in lung extracts, expressed as a percentage of the corresponding saline injections. Bars represent mean values ±SD. Statistically significant differences are indicated by the corresponding t-test p values.

TNF is initially produced as a 26 kDa transmembrane molecule (tmTNF), later to be cleaved by TNF-α Converting Enzyme (TACE) to release the soluble 17 kDa TNF cytokine (solTNF) [20]. While both forms of TNF have been shown to be functionally active, no specific role has been attributed to either one [21]. To discriminate the form(s) of TNF necessary for the development of PF, we administered BLM to genetically modified mice that express only the transmembrane form [22]. Remarkably, although* tnf^tm/tm^* mice developed marked pulmonary inflammation, as assessed by BALF inflammatory cell counts and histopathology (Fig. 1A,B; *tnf^tm/tm^*), they did not overexpress collagen and did not develop any fibrotic lesions (Fig. 1C,A; *tnf^tm/tm^*). To confirm the necessity of solTNF for PF development, a single low dose (200 ng/ml) of aerosolized recombinant human TNF (rhTNF) was administered to *tnf^tm/tm^* mice immediately prior to BLM injection. Reconstituted *tnf^tm/tm^* mice now overexpressed collagen and developed fibrotic lesions (Fig. 2). Therefore, tmTNF-juxtacrine induced inflammation is not sufficient to activate the fibrotic phase of the disease, whereas additional paracrine signaling from the soluble form of TNF is needed.

### Lymphocytes are necessary for solTNF-driven PF

**Figure 3:**
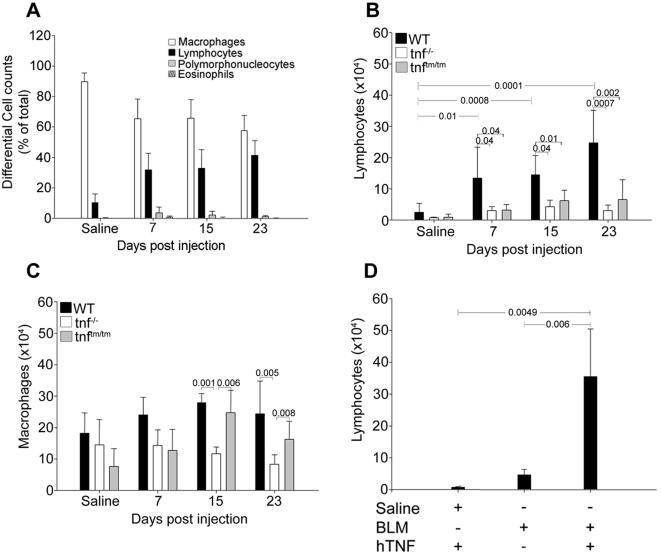
solTNF-dependent lymphocyte infiltration into the lung correlates with BLM/PF development Qualitative assessment of the inflammatory response elicited by BLM in WT and transgenic mice, revealed after differential cell counts of inflammatory cells in BALF. BLM preferentially induces increased infiltration of lymphocytes and subsequent fibrosis only in the presence of solTNF. (A) Different cell populations in the lungs of WT mice expressed as a percentage of total cells. (B) Absolute lymphocyte and (C) macrophage numbers in the lungs of WT, *tnf^−/−^* and *tnf^tm/tm^* mice. (D) Absolute lymphocyte numbers in the lungs of *tnf^tm/tm^* mice 23 days after treatment with hTNF and BLM. Bars represent mean values ±SD. Statistically significant differences are indicated by the corresponding t-test p values.

**Figure 4:**
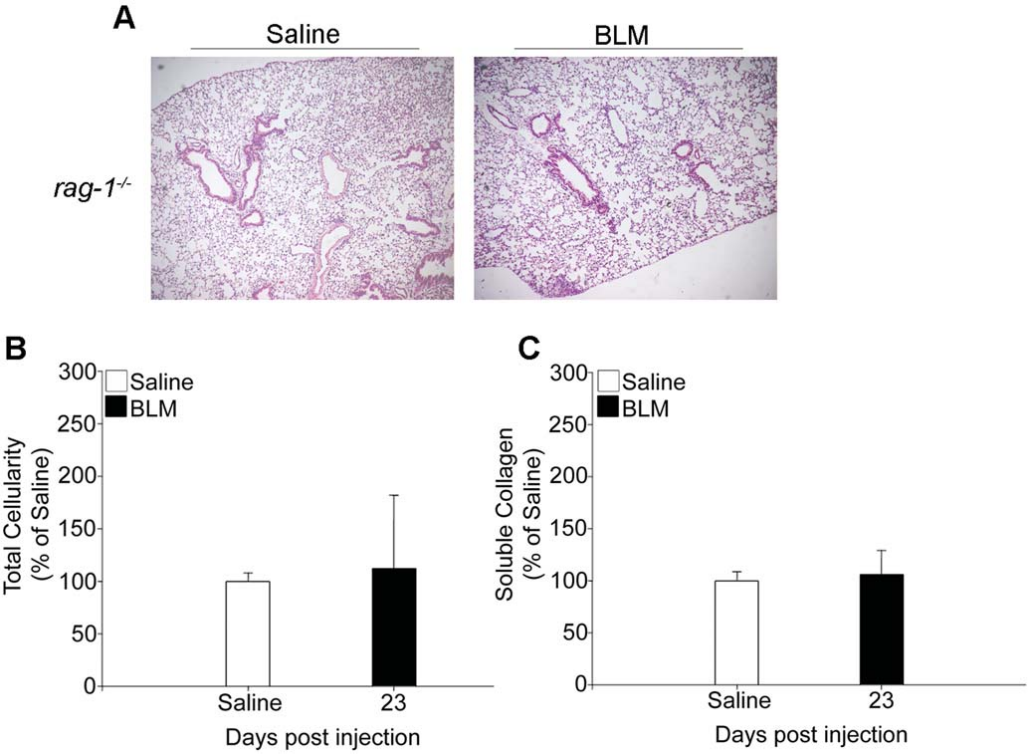
Immunodeficient mice are protected from BLM/PF *rag-1^−/−^* (C57/Bl6) mice that are devoid of T and B cells were injected with BLM and sacrificed 23 days later for disease evaluation. Lack of lymphocytes resulted in failure of disease induction as assessed with (A) representative H/E staining (4×), (B) total inflammatory cell counts in BALF, expressed as a percentage over the corresponding saline injections, (C) soluble collagen determination in lung extracts, expressed as a percentage of the corresponding saline injections. Bars represent the mean values ±SD. Statistically significant differences are indicated by the corresponding t-test p values.

To analyze the TNF-induced inflammatory response, shown to be necessary for the development of PF, the inflammatory cells in BALF were analyzed qualitatively by cytospins. As evident from Figure 3A, under physiological conditions (saline injection) alveolar spaces are populated mainly by macrophages. BLM injection results in a quantitative increase of all inflammatory cells (see also Fig. 1), and in particular to a preferential accumulation of lymphocytes (Fig. 3A,B and C). As expected, BALF from BLM-injected *tnf^−/−^* mice, that failed to develop any inflammatory response and subsequent fibrosis, was almost devoid of any inflammatory cells (Fig. 1 and 3B,C). Remarkably, BALF cellular analysis of BLM-injected *tnf^tmtm^* mice indicated that recruitment of lymphocytes is severely compromised (Fig. 3B), as opposed to the minor differences observed in macrophages (Fig. 3C). Therefore, it seems that solTNF is required for proper recruitment (and/or expansion) of lymphocytes, which in turn are necessary for the development of fibrosis. Indeed, nasal administration of rhTNF to BLM-injected *tnf^tmtm^* mice, that restores disease potential, resulted in massive accumulation of lymphocytes (Fig. 3D). In accordance, administration of an anti-TNF antibody to BLM-injected WT mice resulted in attenuation of inflammatory lymphocytes (unpublished data). To prove further the absolute requirement for lymphocytes in the development of the TNF-driven BLM/PF and resolve the reported discrepancies [23], [24], we administered BLM to *rag-1^−/−^* mice (C57/Bl6) that are devoid of T and B cells [25]. Immunodeficient mice failed to develop any pathological signs, maintaining normal lung architecture (Fig. 4).

### Non-hematopoietic expression of TNF is sufficient for BLM/PF

**Figure 5:**
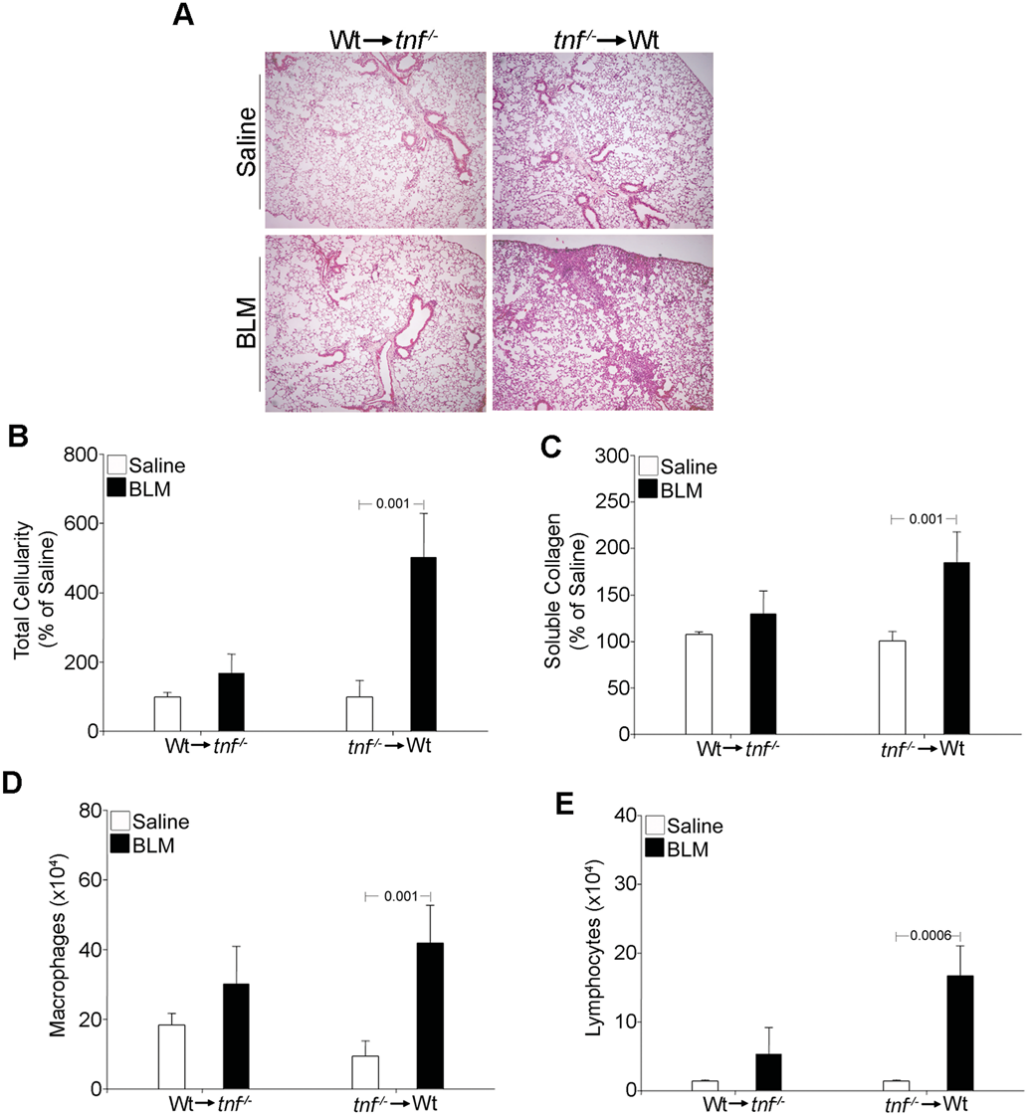
Non-hematopoietic expression of TNF is sufficient for BLM/PF Bone marrow transfer experiments (outlined in Fig. S2) reveal that proper disease development occurs when TNF is produced from radio-resistant non-hematopoietic cells. (A) Representative H/E staining (4×). (B) Total inflammatory cell counts in BALF, expressed as a percentage over the corresponding saline injections. (C) Soluble collagen determination in lung extracts, expressed as a percentage of the corresponding saline injections. (D,E) Absolute numbers of infiltrating macrophages and lymphocytes, respectively. Bars represent the mean values ±SD. Statistically significant differences are indicated by the corresponding t-test p values.

To identify possible cellular TNF sources in PF development, we performed a series of bone marrow transfers into lethally irradiated hosts, as outlined in Figure S2 and described in Materials and Methods. Reconstituted recipient mice bearing hematopoietic cells with the genetic background of the donor mouse and non-hematopoietic cells with the genetic background of the host mouse were then injected with BLM to assess disease development. Mice that lacked TNF expression in the hematopoietic compartment (*tnf^−/−^* to WT) developed both inflammation and fibrosis with proper lymphocyte recruitment; in contrast, abolishing TNF expression from non-hematopoietic cells (WT to *tnf^−/−^*) resulted in complete disease protection (Fig. 5). Thus, these results indicate that radio-resistant non-hematopoietic cells constitute the main cellular source of TNF in the pathogenesis of PF.

**Figure 6:**
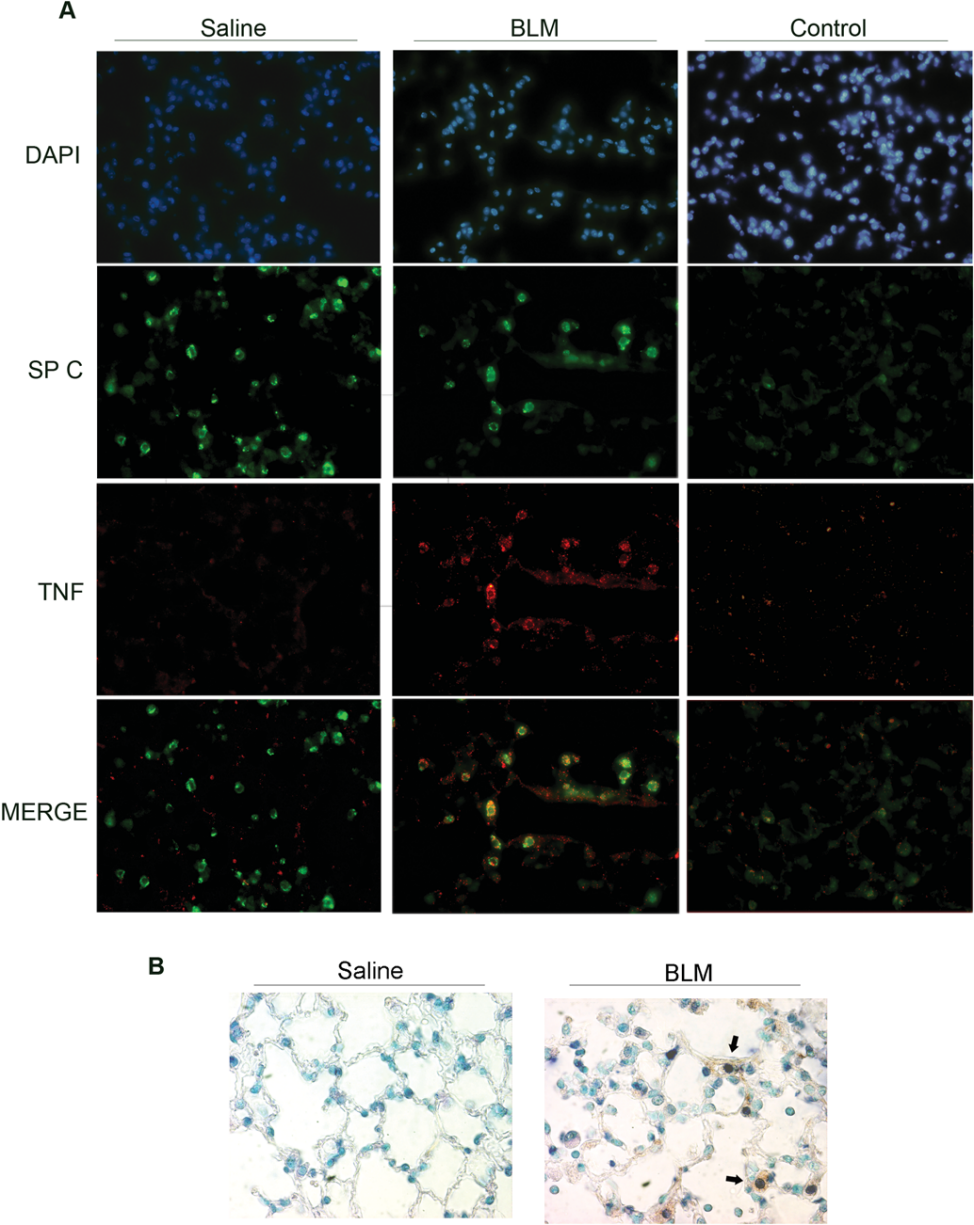
TNF production from apoptosing epithelial cells (A) TNF is expressed from epithelial cells; Colocalization of TNF expression (in red) with an epithelial specific marker (SP C; in green) in lung sections of WT mice 7 days post BLM injection. Control refers to immunostainings without the primary antibody. (B) Simultaneous detection of TNF expression and apoptosis in lung sections of WT mice 7 days post BLM injection. A large number of TUNEL^+^ apoptotic alveolar epithelial cells (blue) exhibit marked TNF expression (in brown; indicated by solid arrow). Sections are counterstained with methyl green and photographed at a magnification of 40×.

To confirm non-hematopoietic TNF production and to identify TNF producing cells, paraffin-embedded lung sections from BLM injected WT mice sacrificed 7 d post injection were immunostained with an anti-TNF antibody. As shown in Figure 6A, TNF expression was localized to the epithelial cells upon injection of BLM. In accordance with the bone marrow transplantation experiments, very few resident macrophages exhibited any significant TNF staining.

BLM-induced DNA double strand breaks in epithelial cells are considered the primary insult in the pathogenesis of BLM/PF, leading to epithelial apoptosis and the initiation of the inflammatory cascade [2], [26]. To examine if BLM-induced TNF-expressing epithelial cells were undergoing apoptosis, anti-TNF-immunostained lung sections were counterstained (TUNEL) for the simultaneous detection of apoptotic cells (Fig. 6B). Indeed, TNF expression colocalized with apoptosing epithelial cells, indicating an early role for TNF production in the pathogenetic cascade.

### TNF expression is necessary for TGF-b1 induction

**Figure 7:**
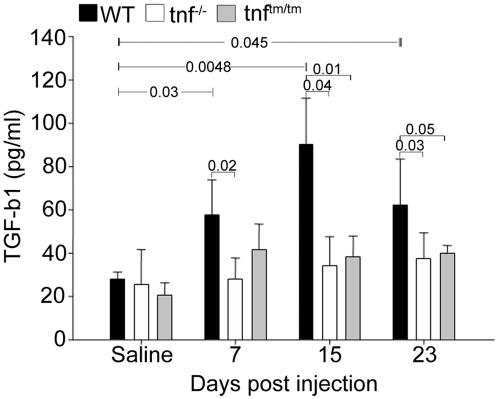
TNF expression is required for TGF-b1 induction TGF-b1 levels determination in BALF samples of the indicated mice with an ELISA assay. Bars represent the mean values ±SD. Statistically significant differences are indicated by the corresponding t-test p values.

TGF-b1, a cytokine long known for its fibrotic properties [27], has been shown to be able to bypass the absolute requirement for TNF signaling in the development of the disease, since TGF-b1 overexpression induces fibrogenesis in the lungs of the fibrogenic-resistant double TNF Receptor (TNFR) deficient mice [28]. Interestingly, in our experimental model, TNF-dependent disease resistance was always associated with diminished TGF-b1 expression (Fig. 7), indicating that TNF signals are required for macrophage/fibroblast production of this major pro-fibrotic cytokine.

### Multiple, redundant TNFR signaling in PF

**Figure 8:**
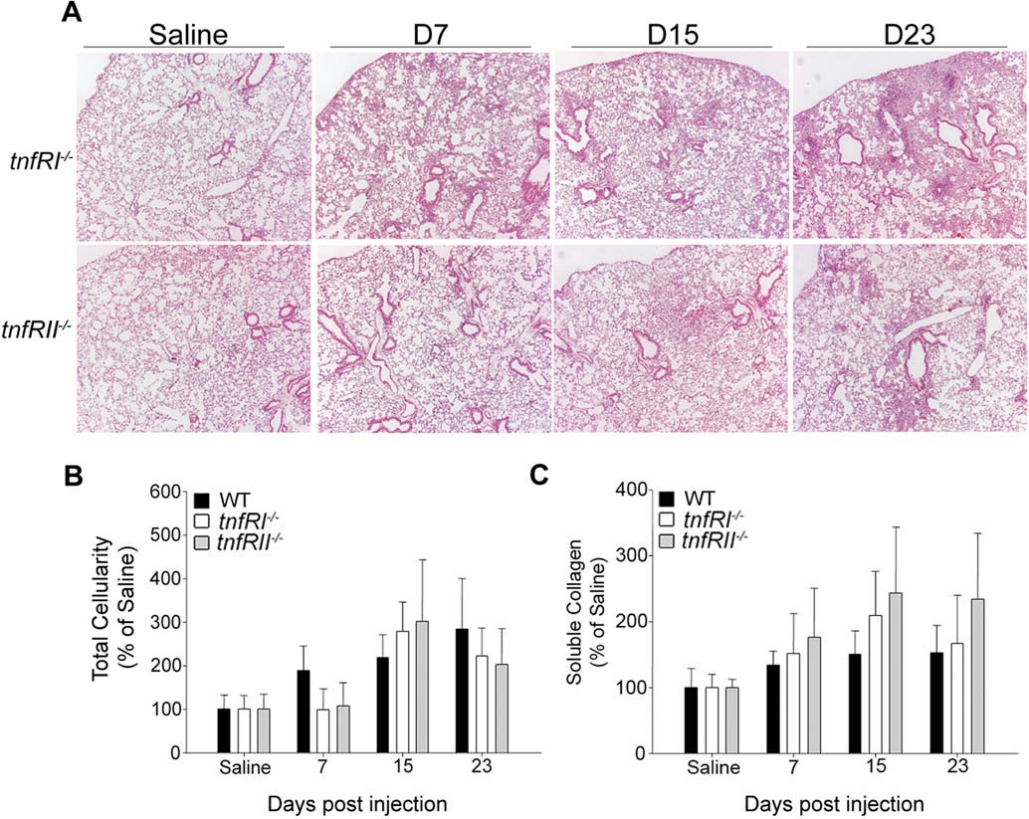
Redundant TNFR signaling in BLM/PF BLM was injected in *tnfRI^−/−^* and *tnfRII^−/−^* mice and disease progression monitored at the indicated time intervals. Both mouse strains developed inflammation and fibrosis as assessed with (A) representative H/E staining (4×), (B) total inflammatory cell counts in BALF, expressed as a percentage over the corresponding saline injections, (C) soluble collagen determination in lung extracts, expressed as a percentage of the corresponding saline injections. Bars represent mean values ±SD. Statistically significant differences are indicated by the corresponding t-test p values.

TNF exerts its effects through two distinct receptors, TNFRI (p55) and TNFRII (p75), expressed in almost all cell types [29]. Although solTNF is regarded as the main ligand for TNFRI and tmTNF is believed to be superior to solTNF in activating TNFRII [30], the precise role of the different TNF receptors is far from solved. Lack of TNFRI & RII receptor signalling protects mice from developing fibrotic lesions upon exposure to asbestos [31]. Similarly, BLM administration to C57/Bl6 mice lacking both TNF receptors also resulted in disease protection, indicating the absolute necessity of TNFR signalling in BLM/PF [32]. To establish TNFR specificity and discriminate receptor usage in the development of PF, BLM was administered to genetically modified mice lacking either receptor (*tnfRI^−/−^* and *tnfRII^−/−^*). Both mouse lines developed both pulmonary inflammation and fibrosis with minor differences from WT mice (Fig. 8), indicating a redundant role of TNF receptors in the development of PF and/or the existence of compensatory mechanisms. In agreement and considering the pleiotropy of TNF and the central role of TNFRI in many biological processes, TNFRI null mice have a remarkably normal development. Similarly and in view of the TNFRII mediated activity *in vitro* in the T-cell compartment, normal development of thymocytes and lymphocytes in TNFRII null mice was quite unexpected [29].

**Figure 9:**
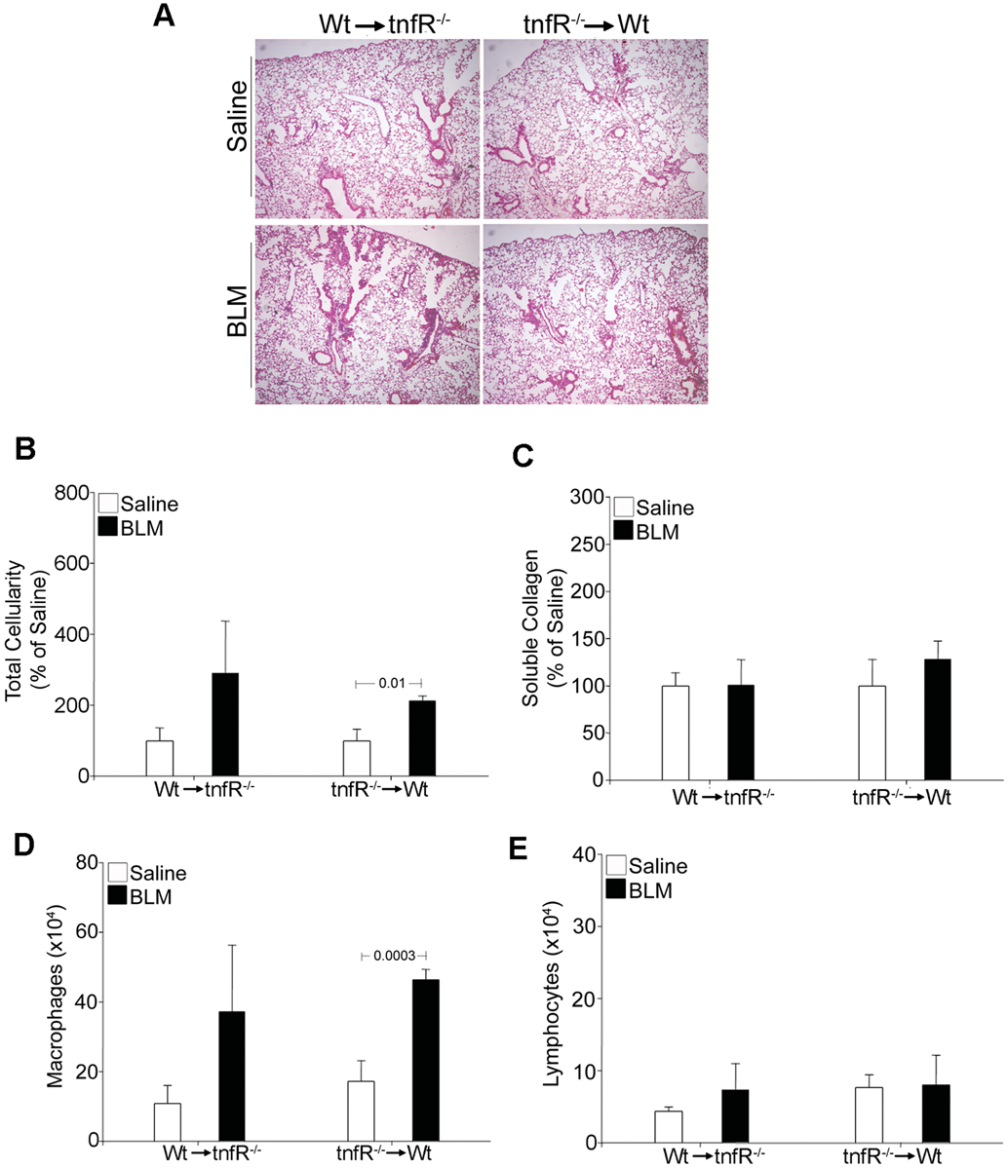
Multiple cellular dependencies on TNFR signaling Bone marrow transfer experiments (outlined in Fig. S2) reveal that proper disease development requires the presence of TNF receptors in both non-hematopoietic and hematopoietic cellular compartments. (A) Representative H/E staining (4×), (B) total inflammatory cell counts in BALF, expressed as a percentage over the corresponding saline injections. (C) Soluble collagen determination in lung extracts, expressed as a percentage over the corresponding saline injections. (D,E) Absolute numbers of infiltrating macrophages and lymphocytes, respectively. Bars represent the mean values ±SD. Statistically significant differences are indicated by the corresponding t-test p values.

Given the TNFR necessity [33] and redundancy (Fig. 8) and in order to identify the TNF responding cellular compartments, mice lacking both TNF receptors (*tnfR^−/−^)* were utilized as donors and/or recipients in bone marrow transfer experiments as above (Outlined in Fig. S2). Mice lacking TNFR signaling in either non-hematopoietic (WT to *tnfR^−/−^*) or hematopoietic (*tnfR^−/−^* to WT) compartments were capable of eliciting an inflammatory response to BLM (Fig. 9 A,B) composed mainly of macrophages (Fig. 9D). However, absence of TNF receptors in either compartment resulted in deficient lymphocyte recruitment and thus protection from the development of fibrosis (Fig. 9A,C,E). Therefore it seems that multiple TNF signals to different cellular compartments are needed for the transition from the inflammatory to the fibrotic phase of the disease.

## Discussion

Wound healing is a fundamental biological process crucial for survival, which allows the ordered replacement of dead or injured cells and the restoration of normal tissue architecture. Tissue damage can result from several acute or chronic stimuli, including infections, autoimmune reactions and mechanical injury. The repair process involves two distinct stages: a regenerative, inflammatory phase, in which the microenvironment attempts to replace injured cells; and a fibrotic phase, in which connective tissue replaces normal parenchymal tissue. However, although initially beneficial, failure to control the healing process can lead to considerable tissue remodeling and the formation of permanent scar tissue. IPF is a prototype fibrotic disease involving abnormal wound healing in response to multiple sites of ongoing alveolar epithelial injury. While the direct involvement of fibrotic mechanisms in the pathogenesis of IPF remains undisputed, the lack of natural history of the disease has created significant controversy on the extent of the contribution of inflammatory mechanisms.

TNF, the major proinflammatory cytokine, was among the first candidate genes to be strongly associated with IPF. Although TNF's involvement in IPF development seems apparent [10]–[19], [34]–[36], a number of studies utilizing genetically modified animals to overexpress and/or ablate TNF in various models of IPF have yielded contradictory and/or inconclusive results. However, these studies were performed in different animals with different genetic backgrounds and ages, while the pathology was induced with a variety of agents (asbestos, silica and BLM), through different routes of administration (intratracheal, intravenous, intraperitoneal and subcutaneous) and different dosing [28], [31], [32], [37]–[43]. To resolve discrepancies and to genetically dissect TNF signaling in the pathogenesis of IPF, we utilized the BLM-induced animal model of PF, the closest equivalent of the human disease. BLM was injected intravenously in order to 1) reproduce the route of administration of the only human PF with a known etiology (i.e., associated with BLM cancer chemotherapy) and 2) to avoid the pronounced and acute effects of the very invasive and inflammation prone intratracheal route of administration. All animals were inbred from the same colony of C57/Bl6 mice for over 20 generations, thus minimizing genetic background effects and enabling direct comparisons. Inflammation and fibrosis were assessed with histopathology, inflammatory cell counts, soluble collagen expression and determination of cytokine levels. Moreover, TNF producing and responding cellular compartments were determined through a series of bone marrow transfer experiments. All results are summarized in Table 1.

**Table 1:**
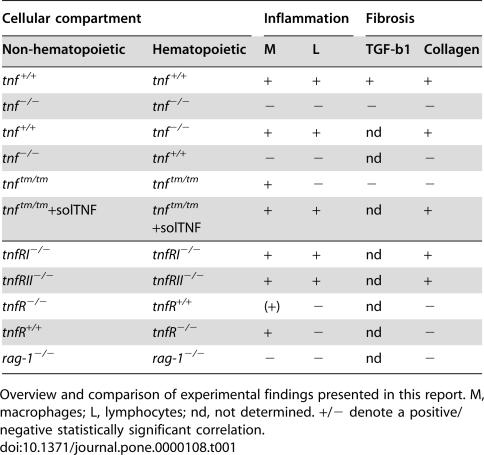
Genetic dissection of TNF signalling in BLM/PF

Cellular compartment		Inflammation		Fibrosis	
Non-hematopoietic	Hematopoietic	M	L	TGF-b1	Collagen
*tnf^+/+^*	*tnf^+/+^*	+	+	+	+
*tnf^−/−^*	*tnf^−/−^*	−	−	−	−
*tnf^+/+^*	*tnf^−/−^*	+	+	nd	+
*tnf^−/−^*	*tnf^+/+^*	−	−	nd	−
*tnf^tm/tm^*	*tnf^tm/tm^*	+	−	−	−
*tnf^tm/tm^*+solTNF	*tnf^tm/tm^*+solTNF	+	+	nd	+
*tnfRI^−/−^*	*tnfRI^−/−^*	+	+	nd	+
*tnfRII^−/−^*	*tnfRII^−/−^*	+	+	nd	+
*tnfR^−/−^*	*tnfR^+/+^*	(+)	−	nd	−
*tnfR^+/+^*	*tnfR^−/−^*	+	−	nd	−
*rag-1^−/−^*	*rag-1^−/−^*	−	−	nd	−

Overview and comparison of experimental findings presented in this report. M, macrophages; L, lymphocytes; nd, not determined. +/− denote a positive/negative statistically significant correlation.

TNF was shown to be a prerequisite for the development of the disease, and *tnf^−/−^* mice were completely protected from both inflammation and fibrosis. For the first time, the necessary TNF expression was shown to originate from the non-hematopoietic compartment, predominately from apoptosing alveolar epithelial cells. In agreement, TNF expression in lung biopsies of IPF human patients was localized mainly in alveolar epithelial cells [11], [12], which, in independent studies, exhibit strong labeling of fragmented DNA [44] and were shown to express a variety of apoptotic proteins and markers [45], [46]. Epithelial cell apoptosis is considered as the initiating pathogenic insult of IPF [2], [26] and its inhibition abrogates the development of BLM/PF [47]–[49]. Moreover, alveolar epithelial apoptosis seems to be a perpetuating event in the pathological cascade of IPF, since apoptotic epithelial cells are detected primarily in areas immediately adjacent to underlying foci of myofibroblasts [50], which have been found to induce alveolar epithelial cell apoptosis in vitro [51], [52]. Therefore TNF expression could trigger not only an initial acute inflammatory response to tissue damage, but could provide chronic inflammatory signals that would exceed the de-activation thresholds of fibrotic mechanisms and lead to uncontrolled wound healing.

TNF, as well as several other growth factors and cytokines, is initially synthesized as a transmembrane molecule, later to be shed by TACE to release the soluble cytokine [20]. Conceivably, given the wide range of pleiotropic biological activities of TNF, surface localization may serve to restrict activity to the local microenvironment, whereas release may lead to distal effects. *tnf^tm/tm^* mice, although they transcribe normal levels of TNF mRNA, express only the transmembrane form of TNF shown to be active and properly regulated [22], [53]. Similar to *tnf^−/−^* mice, *tnf^tm/tm^* mice were resistant to fibrosis induction upon BLM injection. In contrast, tmTNF expression was sufficient to induce inflammation, driven predominantly from macrophages. Complementation with aerosolized rhTNF allowed further recruitment of lymphocytes, overexpression of TGF-b1 and overproduction of collagen. Thus, our findings indicate a different role for the two TNF forms in the pathogenesis of BLM/PF. It appears that tmTNF directly or indirectly controls recruitment of macrophages, while solTNF is needed for lymphocyte recruitment/expansion and the transition from the inflammatory to the fibrotic phase of the disease. In further support of a unique role for tmTNF, it was shown to be sufficient to drive an inflammatory reaction and to provide partial control of *Mycobacterium tuberculosis* and *Listeria monocytogenes* infections [22], [54]–[56]; however, in the absence of solTNF, tmTNF does not support the proper development of rheumatoid arthritis and experimental autoimmune encephalomyelitis [22], [53].

Disease pathogenesis in the BLM/PF model presented here was always associated with preferential accumulation of lymphocytes (Fig. 3A), implying participation of lymphocytes in the pathogenesis of PF in good agreement with a number of previous reports [24], [57]–[60]. In sharp contrast, it has been reported that BLM induced PF is independent of lymphocytes, since immunodeficient SCID mice [61] were shown not to be protected from BLM [23]. Given that SCID mice are leaky, containing a small number of lymphocytes, and in order to resolve discrepancies, BLM was administrated to *rag-1^−/−^* mice which are completely devoid of T&B cells [25]. *rag-1^−/−^* immunodeficient mice were shown to be completely protected from PF development (Fig. 4). Additional experimentation will be required to further understand the involvement of the acquired immune system in the pathogenesis of BLM induced PF and human IPF. Experimental and clinical studies suggest that a persistent imbalance in the expression of Th2 versus Th1 cytokines in the lung represents an additional possible mechanism for the progression of pulmonary fibrosis. Although corticosteroids and immunosuppressants failed to cure the disease, promising results have been obtained from clinical trials with IFN-γ1b [62], [63].

In addition to the well-documented capacity of TNF to activate non-specific or innate inflammation, ample evidence indicates that TNF may also affect the adaptive immune response. TNF has been shown to induce thymocyte proliferation, to act as mitogen for activated αβ T cells or unstimulated γδ T cells, and has been identified as a crucial factor in the recruitment and activation of antigen-presenting cells, thereby enhancing T cell activation in situ [64]. On the contrary and as expected for a pleiotropic cytokine, TNF can also mediate mature T cell-receptor-induced apoptosis through TNFRII [65]. As shown in the present report, the observed protection in *tnf^−/−^* and *tnf^tm/tm^* mice was always correlated with diminished lymphocyte accumulation (Fig. 3B,C). More importantly, complementation of *tnf^tm/tm^* mice with soluble rhTNF that restored disease potential resulted in massive accumulation of lymphocytes (Fig. 3D). Thus, it seems that soluble TNF is required for appropriate lymphocyte accumulation and/or expansion, which can in turn mediate the transition to the fibrotic phase of the disease. In accordance, forced transgenic expression (constitutive or inducible) of TNF from type II epithelial cells through transgenic genetic modifications, resulted in leukocyte infiltration with a predominance of lymphocytes. This led to subsequent enlargement of air spaces in association with alveolar wall thickening due to increased accumulation of collagen [41], [43]. Similarly, systemic hTNF overexpression in the Tg3647 transgenic mouse model of rheumatoid arthritis [66], results in massive lymphocyte infiltration in the lung (unpublished data). Interestingly, unlike with the epithelial-specific TNF expression, systemic, non-epithelial overexpression of TNF results in pulmonary pathology characterized as lymphoid interstitial pneumonitis, exhibiting organized follicles that stained positively for both B and T cells (unpublished data). Bone marrow transfer experiments from *tnfR^−/−^* mice and vice versa indicate that lymphocyte recruitment and disease induction requires the presence of TNF receptors in both cellular compartments, suggesting that TNF-mediated lymphocyte recruitment is indirect, most likely involving non-hematopoietic cells as previously implied [67].

Absence of TNF-mediated lymphocyte recruitment (and consequent disease induction) was always associated with lack of TGF-b1 expression (Fig. 7). TGF-b1 is largely responsible for fibroblast activation/differentiation, as well as collagen overexpression [27]. Moreover, in our experimental model diminished TGF-b1 expression correlated with diminished Tissue Inhibitor of Metalloproteinase 1 (TIMP1) expression and MMP9/TIMP1 reversal of expression ratio, towards a non-degrading environment (Fig. S3). TGF-b1 conditional overexpression induces fibrosis even in the absence of inflammation and TNF expression [68]. As shown recently, TGF-b1 induction from macrophages occurs in a two-stage process requiring both TNF and IL-13 [69]. IL-13, a prototype Th2 cytokine, is a major inducer of fibrosis in many chronic infectious and autoimmune diseases [70], [71], while TNF has been shown to be a critical component of the IL-13 mediated protective Th2 response during helminth infection [72]. Therefore, since there is no evidence of TGF-b1 induction in the absence of inflammation, coordinated signaling of TNF/IL-13 could control induction of this major profibrotic cytokine and the transition to the fibrotic phase of the disease.

IPF and related pulmonary fibrotic disorders affect millions of individuals worldwide, where the clinical outcome for the vast majority of the patients remains poor. Administration of corticosteroids and other general anti-inflammatory and immunosuppressant drugs fails to control the disease, leading physicians to alternate modes of treatment. Surprisingly, there have been only a few isolated case reports in the literature accessing the efficacy of anti-TNF treatment, perhaps due to the failure of corticosteroids as well as to the increased risk of infections associated with current anti-TNF biologics. Treatment with infliximab (chimeric anti-TNF antibody) of a rheumatoid arthritis patient with progressive pulmonary symptoms associated with radiographic fibrosis in order to control articular symptoms resulted in sustained improvement in dyspnea, cough and exercise tolerance in addition to improvement of joint symptoms [73]. Similarly, infliximab treatment of a patient with lung fibrosis and pulmonary hypertension associated with advanced systemic sclerosis refractory to conventional therapies seemed effective, improving quality of life [74]. Moreover, etanercept (a hybrid molecule consisting of TNFRII linked to the Fc domain of human IgG1) administration in nine subjects with IPF demonstrated tolerability, with functional improvement in some [75].

Our results presented in this report reinforce the importance of assessing the efficacy of TNF antagonists in IPF treatment. More importantly, specific blockade of soluble TNF would be predicted to abolish the detrimental pro-fibrotic effects of TNF, while maintaining sufficient host defense against infections. Preliminary results from on-going *in vivo* studies with an engineered dominant-negative peptide acting as a selective inhibitor of soluble TNF [76] indicated comparable efficacy with etanercept, which alleviated pathological symptoms as previously reported [14], [18], [35]. Although anti-TNF treatment is unlikely to cure the disease (as in the case of rheumatoid arthritis and Crohn's disease), it would still offer symptomatic relief and quality of life improvement.

## Materials and Methods

### Animals

The generation of *tnf^−/−^* [77], *tnf^tm/tm^* [22], *tnfRI^−/−^* [78], *tnfRII^−/−^* [79], and *rag-1^−/−^* [25] mice by homologous recombination in ES cells has been described previously. All mice strains were bred and maintained in the C57/Bl6 background (same colony) for over 20 generations in the animal facilities of the Biomedical Sciences Research Center “Alexander Fleming” under specific pathogen-free conditions, in compliance with the Declaration of Helsinki principles. Mice were housed at 20–22°C, 55±5% humidity, and a 12 h light-dark cycle; food and water was given ad libitum. All experimentation was approved by an internal Institutional Review Board, as well as by the veterinary service and fishery department of the local governmental prefecture.

### Animal model of BLM/PF

PF was induced by a single tail vein injection of Bleomycin hydrogen chloride (100 mg/kg body weight; 1/3 LD50; Nippon Kayaku Co. Ltd., Tokyo) to 6- to 8-wk-old mice as previously reported [80]–[82] and as described in detail in Figure S1. Disease progression was monitored at 7, 15 and 23 days after Bleomycin injection, in parallel with control littermate mice injected similarly with saline (only at 23 d post injection). At the appropriate endpoints mice were weighed and sacrificed; their lungs were lavaged three times with 1.0 ml aliquots of saline (BronchoAlveolar Lavage Fluid; BALF), followed by perfusion through the right ventricle of the heart with 10 ml of PBS. The lungs were then removed, weighed and dissected as indicated in Figure S1. Total and differential cell counts were assessed with Trypan Blue and May-Giemsa staining respectively; collagen levels were quantified with the Sircol collagen assay (Biocolor Ltd., Belfast, UK) essentially as previously reported [83] and as described in detail in Figure S1. All values were normalized over control injections, in order to be able to compare different mouse strains. The left lung was divided sagittally; one half was snap-frozen in liquid N_2_ for RNA extraction while the other half was used for histopathology, where paraffin-embedded lung tissue samples were sectioned and stained with Haematoxylin/Eosin and/or Masson trichromy (Fig. S1).

### Bone marrow transplantation

Bone marrow from 8-wk-old mutant and wild-type mice (C57/Bl6) was obtained from femurs and tibia, essentially as previously described [84] and as outlined in Figure S2. 10^7^ bone marrow cells in 200 µl of HBSS were injected intravenously into lethally irradiated (980 rad) recipient mice (C57/Bl6), a dosage that never produced any signs of inflammation or fibrosis in control animals (as it can be seen in the control groups of Figs. 5 and 9). Mice were maintained in isolated specific pathogen-free conditions and kept on an antibiotic regime (neomycin sulfate 1 mg/ml) for 2 wk. Reconstitution was assessed via specific staining for CD4/CD8, B220 and GR-1 antigens via FACS® analysis of heparinized blood samples (Fig S2). After durable bone marrow engraftment had been established (8 wk), pulmonary fibrosis was induced by administration of bleomycin. Mice were sacrificed 23 d post-injection and lung injury was assessed as above.

### Immunohistochemistry and TUNEL assay

Deparaffinized and rehydrated 5 µm-thick sections of paraffin embedded lung tissue samples were incubated with a goat polyclonal anti-mouse TNF Ab (R&D Systems, Minneapolis, MN, USA) overnight at 4°C. Sections were then incubated with 0.03% H_2_O_2_ followed by anti-goat IgG-HRP (Vector Laboratories, Burlingame, CA, USA) for 30 min at RT. Bound peroxidase activity was detected by staining with diaminobenzidine (DAB, Sigma, St Louis, MO, USA). For the simultaneous in situ detection of apoptotic cells (TUNEL assay), sections were first processed as above and after color development with DAB, they were incubated with terminal deoxynucleotidyl transferase (TdT) (Roche Diagnostics, Mannheim, Germany) at 37^o^C for 1 h in a humidified chamber. Then, according to manufacturer's instructions, sections were incubated with the alkaline phosphatase-conjugated secondary Ab and developed using the Fast Blue kit (Vector Laboratories, Burlingame, CA, USA). Prior to mounting, sections were counterstained with methyl green.

For the identification of TNF producing cells, lung sections processed as above were incubated with a goat polyclonal anti-mouse TNF Ab (R&D Systems, Minneapolis, MN, USA) for 1 h at RT. After washing, sections were incubated with a donkey anti-goat Alexa Fluor 555 secondary Ab (Molecular Probes/Invitrogen, Carlsbad, CA, USA) for 30 min at RT. Sections were then sequentially incubated with an anti- pro-SP-C Ab (Upstate, Temecula, CA, USA) at RT for 1 h, followed by a goat anti-rabbit Alexa Fluor 488 Ab (Molecular Probes/Invitrogen, Carlsbad, CA, USA). Finally, sections were mounted and photographed under a Nikon ECLIPSE E800 microscope (Nikon Corp., Shinagawa-ku, Tokyo, Japan).

### Airway challenge with rhTNF

Recombinant human TNF (hTNF; XENCOR, CA, USA) was dissolved at a concentration of 200 ng/ml in normal saline, at a final volume of 3 ml. The solution was administered by a custom made nebulizer flowing at 4 L/min of O_2_ for 25 min into an airtight chamber containing 5–7 mice.

### ELISA

Quantification of TGF-b1 in BALF samples was performed with the Quantikine Immunoassay, according to manufacturer's instructions (R&D systems, Inc, Minneapolis, MN, USA).

### Statistical analysis

BALF cell counts, collagen levels and ELISA values were analyzed for statistical significance with student's t-test (in pairwise comparisons), utilizing the statistical software package Sigmaplot (Systat Software Inc, Richmond, CA, USA).

## Supporting Information

Figure S1 Animal model of BLM-induced Pulmonary Fibrosis.
(0.23 MB DOC)

Figure S2 Schematic overview of bone marrow transplantation experiments.
(1.62 MB TIF)

Figure S3 solTNF but not tmTNF can support a non-degrading, profibrogenic environment.
(0.19 MB TIF)

## References

[1] Gross TJ, Hunninghake GW (2001). Idiopathic pulmonary fibrosis.. N Engl J Med.

[2] Selman M, King TE, Pardo A (2001). Idiopathic pulmonary fibrosis: prevailing and evolving hypotheses about its pathogenesis and implications for therapy.. Ann Intern Med.

[3] Gauldie J (2002). Pro: Inflammatory mechanisms are a minor component of the pathogenesis of idiopathic pulmonary fibrosis.. Am J Respir Crit Care Med.

[4] Gauldie J, Kolb M, Sime PJ (2002). A new direction in the pathogenesis of idiopathic pulmonary fibrosis?. Respir Res.

[5] Sheppard D (2001). Pulmonary fibrosis: a cellular overreaction or a failure of communication?. J Clin Invest.

[6] Strieter RM (2001). Mechanisms of pulmonary fibrosis: conference summary.. Chest.

[7] Strieter RM (2002). Con: Inflammatory mechanisms are not a minor component of the pathogenesis of idiopathic pulmonary fibrosis.. Am J Respir Crit Care Med.

[8] Kollias G (2004). Modeling the function of tumor necrosis factor in immune pathophysiology.. Autoimmun Rev.

[9] Locksley RM, Killeen N, Lenardo MJ (2001). The TNF and TNF receptor superfamilies: integrating mammalian biology.. Cell.

[10] Hasegawa M, Fujimoto M, Kikuchi K, Takehara K (1997). Elevated serum tumor necrosis factor-alpha levels in patients with systemic sclerosis: association with pulmonary fibrosis.. J Rheumatol.

[11] Nash JR, McLaughlin PJ, Butcher D, Corrin B (1993). Expression of tumour necrosis factor-alpha in cryptogenic fibrosing alveolitis.. Histopathology.

[12] Piguet PF, Ribaux C, Karpuz V, Grau GE, Kapanci Y (1993). Expression and localization of tumor necrosis factor-alpha and its mRNA in idiopathic pulmonary fibrosis.. Am J Pathol.

[13] Ziegenhagen MW, Schrum S, Zissel G, Zipfel PF, Schlaak M (1998). Increased expression of proinflammatory chemokines in bronchoalveolar lavage cells of patients with progressing idiopathic pulmonary fibrosis and sarcoidosis.. J Investig Med.

[14] Piguet PF, Collart MA, Grau GE, Kapanci Y, Vassalli P (1989). Tumor necrosis factor/cachectin plays a key role in bleomycin-induced pneumopathy and fibrosis.. J Exp Med.

[15] Thrall RS, Vogel SN, Evans R, Shultz LD (1997). Role of tumor necrosis factor-alpha in the spontaneous development of pulmonary fibrosis in viable motheaten mutant mice.. Am J Pathol.

[16] Phan SH, Kunkel SL (1992). Lung cytokine production in bleomycin-induced pulmonary fibrosis.. Exp Lung Res.

[17] Ortiz LA, Lasky J, Hamilton RF, Holian A, Hoyle GW (1998). Expression of TNF and the necessity of TNF receptors in bleomycin-induced lung injury in mice.. Exp Lung Res.

[18] Piguet PF, Collart MA, Grau GE, Sappino AP, Vassalli P (1990). Requirement of tumour necrosis factor for development of silica-induced pulmonary fibrosis.. Nature.

[19] Whyte M, Hubbard R, Meliconi R, Whidborne M, Eaton V (2000). Increased risk of fibrosing alveolitis associated with interleukin-1 receptor antagonist and tumor necrosis factor-alpha gene polymorphisms.. Am J Respir Crit Care Med.

[20] Moss ML, Jin SL, Milla ME, Bickett DM, Burkhart W (1997). Cloning of a disintegrin metalloproteinase that processes precursor tumour-necrosis factor-alpha.. Nature.

[21] Kriegler M, Perez C, DeFay K, Albert I, Lu SD (1988). A novel form of TNF/cachectin is a cell surface cytotoxic transmembrane protein: ramifications for the complex physiology of TNF.. Cell.

[22] Alexopoulou L, Kranidioti K, Xanthoulea S, Denis M, Kotanidou (2006). Transmembrane TNF protects mutant mice against intracellular bacterial infections, chronic inflammation and autoimmunity.. Eur J Immunol.

[23] Helene M, Lake-Bullock V, Zhu J, Hao H, Cohen DA (1999). T cell independence of bleomycin-induced pulmonary fibrosis.. J Leukoc Biol.

[24] Huaux F, Liu T, McGarry B, Ullenbruch M, Xing Z (2003). Eosinophils and T lymphocytes possess distinct roles in bleomycin-induced lung injury and fibrosis.. J Immunol.

[25] Mombaerts P, Iacomini J, Johnson RS, Herrup K, Tonegawa S (1992). RAG-1-deficient mice have no mature B and T lymphocytes.. Cell.

[26] Allen JT, Spiteri MA (2002). Growth factors in idiopathic pulmonary fibrosis: relative roles.. Respir Res.

[27] Mauviel A (2005). Transforming growth factor-beta: a key mediator of fibrosis.. Methods Mol Med.

[28] Liu JY, Sime PJ, Wu T, Warshamana GS, Pociask D (2001). Transforming growth factor-beta(1) overexpression in tumor necrosis factor-alpha receptor knockout mice induces fibroproliferative lung disease.. Am J Respir Cell Mol Biol.

[29] Vandenabeele P, Declercq W, Beyaert R, Fiers W (1995). Two tumour necrosis factor receptors: structure and function.. Trends Cell Biol.

[30] Grell M, Douni E, Wajant H, Lohden M, Clauss M (1995). The transmembrane form of tumor necrosis factor is the prime activating ligand of the 80 kDa tumor necrosis factor receptor.. Cell.

[31] Liu JY, Brass DM, Hoyle GW, Brody AR (1998). TNF-alpha receptor knockout mice are protected from the fibroproliferative effects of inhaled asbestos fibers.. Am J Pathol.

[32] Ortiz LA, Lasky J, Lungarella G, Cavarra E, Martorana P (1999). Upregulation of the p75 but not the p55 TNF-alpha receptor mRNA after silica and bleomycin exposure and protection from lung injury in double receptor knockout mice.. Am J Respir Cell Mol Biol.

[33] Ortiz LA, Lasky JA, Safah H, Reyes M, Miller A (1999). Exacerbation of bleomycin-induced lung injury in mice by amifostine.. Am J Physiol.

[34] Kapanci Y, Desmouliere A, Pache JC, Redard M, Gabbiani G (1995). Cytoskeletal protein modulation in pulmonary alveolar myofibroblasts during idiopathic pulmonary fibrosis. Possible role of transforming growth factor beta and tumor necrosis factor alpha.. Am J Respir Crit Care Med.

[35] Piguet PF, Vesin C (1994). Treatment by human recombinant soluble TNF receptor of pulmonary fibrosis induced by bleomycin or silica in mice.. Eur Respir J.

[36] Zhang K, Gharaee-Kermani M, McGarry B, Remick D, Phan SH (1997). TNF-alpha-mediated lung cytokine networking and eosinophil recruitment in pulmonary fibrosis.. J Immunol.

[37] Fujita M, Shannon JM, Irvin CG, Fagan KA, Cool C (2001). Overexpression of tumor necrosis factor-alpha produces an increase in lung volumes and pulmonary hypertension.. Am J Physiol Lung Cell Mol Physiol.

[38] Fujita M, Shannon JM, Morikawa O, Gauldie J, Hara N (2003). Overexpression of tumor necrosis factor-alpha diminishes pulmonary fibrosis induced by bleomycin or transforming growth factor-beta.. Am J Respir Cell Mol Biol.

[39] Kuroki M, Noguchi Y, Shimono M, Tomono K, Tashiro T (2003). Repression of bleomycin-induced pneumopathy by TNF.. J Immunol.

[40] Lundblad LK, Thompson-Figueroa J, Leclair T, Sullivan MJ, Poynter ME (2005). TNF-{alpha} Over-expression in Lung Disease: a Single Cause Behind a Complex Phenotype.. Am J Respir Crit Care Med.

[41] Miyazaki Y, Araki K, Vesin C, Garcia I, Kapanci Y (1995). Expression of a tumor necrosis factor-alpha transgene in murine lung causes lymphocytic and fibrosing alveolitis. A mouse model of progressive pulmonary fibrosis.. J Clin Invest.

[42] Sime PJ, Marr RA, Gauldie D, Xing Z, Hewlett BR (1998). Transfer of tumor necrosis factor-alpha to rat lung induces severe pulmonary inflammation and patchy interstitial fibrogenesis with induction of transforming growth factor-beta1 and myofibroblasts.. Am J Pathol.

[43] Vuillemenot BR, Rodriguez JF, Hoyle GW (2004). Lymphoid tissue and emphysema in the lungs of transgenic mice inducibly expressing tumor necrosis factor-alpha.. Am J Respir Cell Mol Biol.

[44] Kuwano K, Kunitake R, Kawasaki M, Nomoto Y, Hagimoto N (1996). P21Waf1/Cip1/Sdi1 and p53 expression in association with DNA strand breaks in idiopathic pulmonary fibrosis.. Am J Respir Crit Care Med.

[45] Maeyama T, Kuwano K, Kawasaki M, Kunitake R, Hagimoto N (2001). Upregulation of Fas-signalling molecules in lung epithelial cells from patients with idiopathic pulmonary fibrosis.. Eur Respir J.

[46] Plataki M, Koutsopoulos AV, Darivianaki K, Delides G, Siafakas NM (2005). Expression of apoptotic and antiapoptotic markers in epithelial cells in idiopathic pulmonary fibrosis.. Chest.

[47] Kuwano K, Hagimoto N, Kawasaki M, Yatomi T, Nakamura N (1999). Essential roles of the Fas-Fas ligand pathway in the development of pulmonary fibrosis.. J Clin Invest.

[48] Kuwano K, Kunitake R, Maeyama T, Hagimoto N, Kawasaki M (2001). Attenuation of bleomycin-induced pneumopathy in mice by a caspase inhibitor.. Am J Physiol Lung Cell Mol Physiol.

[49] Wang R, Ibarra-Sunga O, Verlinski L, Pick R, Uhal BD (2000). Abrogation of bleomycin-induced epithelial apoptosis and lung fibrosis by captopril or by a caspase inhibitor.. Am J Physiol Lung Cell Mol Physiol.

[50] Uhal BD, Joshi I, Hughes WF, Ramos C, Pardo A (1998). Alveolar epithelial cell death adjacent to underlying myofibroblasts in advanced fibrotic human lung.. Am J Physiol.

[51] Uhal BD, Joshi I, True AL, Mundle S, Raza A (1995). Fibroblasts isolated after fibrotic lung injury induce apoptosis of alveolar epithelial cells in vitro.. Am J Physiol.

[52] Wang R, Ramos C, Joshi I, Zagariya A, Pardo A (1999). Human lung myofibroblast-derived inducers of alveolar epithelial apoptosis identified as angiotensin peptides.. Am J Physiol.

[53] Ruuls SR, Hoek RM, Ngo VN, McNeil T, Lucian LA (2001). Membrane-bound TNF supports secondary lymphoid organ structure but is subservient to secreted TNF in driving autoimmune inflammation.. Immunity.

[54] Fremond C, Allie N, Dambuza I, Grivennikov SI, Yeremeev V (2005). Membrane TNF confers protection to acute mycobacterial infection.. Respir Res.

[55] Saunders BM, Tran S, Ruuls S, Sedgwick JD, Briscoe H (2005). Transmembrane TNF is sufficient to initiate cell migration and granuloma formation and provide acute, but not long-term, control of Mycobacterium tuberculosis infection.. J Immunol.

[56] Torres D, Janot L, Quesniaux VF, Grivennikov SI, Maillet I (2005). Membrane tumor necrosis factor confers partial protection to Listeria infection.. Am J Pathol.

[57] Hao Z, Hampel B, Yagita H, Rajewsky K (2004). T cell-specific ablation of Fas leads to Fas ligand-mediated lymphocyte depletion and inflammatory pulmonary fibrosis.. J Exp Med.

[58] Huaux F, Liu T, McGarry B, Ullenbruch M, Phan SH (2003). Dual roles of IL-4 in lung injury and fibrosis.. J Immunol.

[59] Izbicki G, Segel MJ, Christensen TG, Conner MW, Breuer R (2002). Time course of bleomycin-induced lung fibrosis.. Int J Exp Pathol.

[60] Zhu J, Cohen DA, Goud SN, Kaplan AM (1996). Contribution of T lymphocytes to the development of bleomycin-induced pulmonary fibrosis.. Ann N Y Acad Sci.

[61] Vladutiu AO (1993). The severe combined immunodeficient (SCID) mouse as a model for the study of autoimmune diseases.. Clin Exp Immunol.

[62] Antoniou KM, Nicholson AG, Dimadi M, Malagari K, Latsi P (2006). Long term clinical effects of IFN-{gamma}-1b and colchicine in idiopathic pulmonary fibrosis.. Eur Respir J.

[63] Nathan SD, Barnett SD, Moran B, Helman DL, Nicholson K (2004). Interferon gamma-1b as therapy for idiopathic pulmonary fibrosis. An intrapatient analysis.. Respiration.

[64] Kollias G, Douni E, Kassiotis G, Kontoyiannis D (1999). On the role of tumor necrosis factor and receptors in models of multiorgan failure, rheumatoid arthritis, multiple sclerosis and inflammatory bowel disease.. Immunol Rev.

[65] Zheng L, Fisher G, Miller RE, Peschon J, Lynch DH (1995). Induction of apoptosis in mature T cells by tumour necrosis factor.. Nature.

[66] Keffer J, Probert L, Cazlaris H, Georgopoulos S, Kaslaris E (1991). Transgenic mice expressing human tumour necrosis factor: a predictive genetic model of arthritis.. Embo J.

[67] Ohmori Y, Wyner L, Narumi S, Armstrong D, Stoler M (1993). Tumor necrosis factor-alpha induces cell type and tissue-specific expression of chemoattractant cytokines in vivo.. Am J Pathol.

[68] Hardie WD, Le Cras TD, Jiang K, Tichelaar JW, Azhar M (2004). Conditional expression of transforming growth factor-alpha in adult mouse lung causes pulmonary fibrosis.. Am J Physiol Lung Cell Mol Physiol.

[69] Fichtner-Feigl S, Strober W, Kawakami K, Puri RK, Kitani A (2006). IL-13 signaling through the IL-13alpha2 receptor is involved in induction of TGF-beta1 production and fibrosis.. Nat Med.

[70] Lee CG, Homer RJ, Zhu Z, Lanone S, Wang X (2001). Interleukin-13 induces tissue fibrosis by selectively stimulating and activating transforming growth factor beta(1).. J Exp Med.

[71] Wynn TA (2003). IL-13 effector functions.. Annu Rev Immunol.

[72] Artis D, Humphreys NE, Bancroft AJ, Rothwell NJ, Potten CS (1999). Tumor necrosis factor alpha is a critical component of interleukin 13-mediated protective T helper cell type 2 responses during helminth infection.. J Exp Med.

[73] Vassallo R, Matteson E, Thomas CF (2002). Clinical response of rheumatoid arthritis-associated pulmonary fibrosis to tumor necrosis factor-alpha inhibition.. Chest.

[74] Bargagli E, Galeazzi M, Bellisai F, Volterrani L, Rottoli P (2005). Infliximab Treatment in a Patient with Systemic Sclerosis Associated with Lung Fibrosis and Pulmonary Hypertension.. Respiration.

[75] Niden A, Koss M, Boylen CT, Wilcox A (2002). An open-label pilot study to determine the potential efficacy of TNFR: FC (Enbrel, etanercept) in the treatment of usual interstitial pneumonitis (UIP).. Am J Respir Crit Care Med.

[76] Steed PM, Tansey MG, Zalevsky J, Zhukovsky EA, Desjarlais JR (2003). Inactivation of TNF signaling by rationally designed dominant-negative TNF variants.. Science.

[77] Pasparakis M, Alexopoulou L, Episkopou V, Kollias G (1996). Immune and inflammatory responses in TNF alpha-deficient mice: a critical requirement for TNF alpha in the formation of primary B cell follicles, follicular dendritic cell networks and germinal centers, and in the maturation of the humoral immune response.. J Exp Med.

[78] Rothe J, Lesslauer W, Lotscher H, Lang Y, Koebel P (1993). Mice lacking the tumour necrosis factor receptor 1 are resistant to TNF-mediated toxicity but highly susceptible to infection by Listeria monocytogenes.. Nature.

[79] Erickson SL, de Sauvage FJ, Kikly K, Carver-Moore K, Pitts-Meek S (1994). Decreased sensitivity to tumour-necrosis factor but normal T-cell development in TNF receptor-2-deficient mice.. Nature.

[80] Hoyt DG, Lazo JS (1992). Murine strain differences in acute lung injury and activation of poly(ADP-ribose) polymerase by in vitro exposure of lung slices to bleomycin.. Am J Respir Cell Mol Biol.

[81] Okudela K, Ito T, Mitsui H, Hayashi H, Udaka N (1999). The role of p53 in bleomycin-induced DNA damage in the lung. A comparative study with the small intestine.. Am J Pathol.

[82] Piguet PF, Vesin C (1994). Pulmonary platelet trapping induced by bleomycin: correlation with fibrosis and involvement of the beta 2 integrins.. Int J Exp Pathol.

[83] Phillips RJ, Burdick MD, Hong K, Lutz MA, Murray LA (2004). Circulating fibrocytes traffic to the lungs in response to CXCL12 and mediate fibrosis.. J Clin Invest.

[84] Kontoyiannis D, Boulougouris G, Manoloukos M, Armaka M, Apostolaki M (2002). Genetic dissection of the cellular pathways and signaling mechanisms in modeled tumor necrosis factor-induced Crohn's-like inflammatory bowel disease.. J Exp Med.

