# Supplementary material for: Soluble TNF Mediates the Transition from Pulmonary Inflammation to Fibrosis
Source: PLoS One. 2006 Dec 27;1(1):e108. doi: 10.1371/journal.pone.0000108 (PMC1762410; doi:10.1371/journal.pone.0000108)
Supplement: Figure S1 — Animal model of BLM-induced Pulmonary Fibrosis. (0.23 MB DOC) [file pone.0000108.s001.doc]

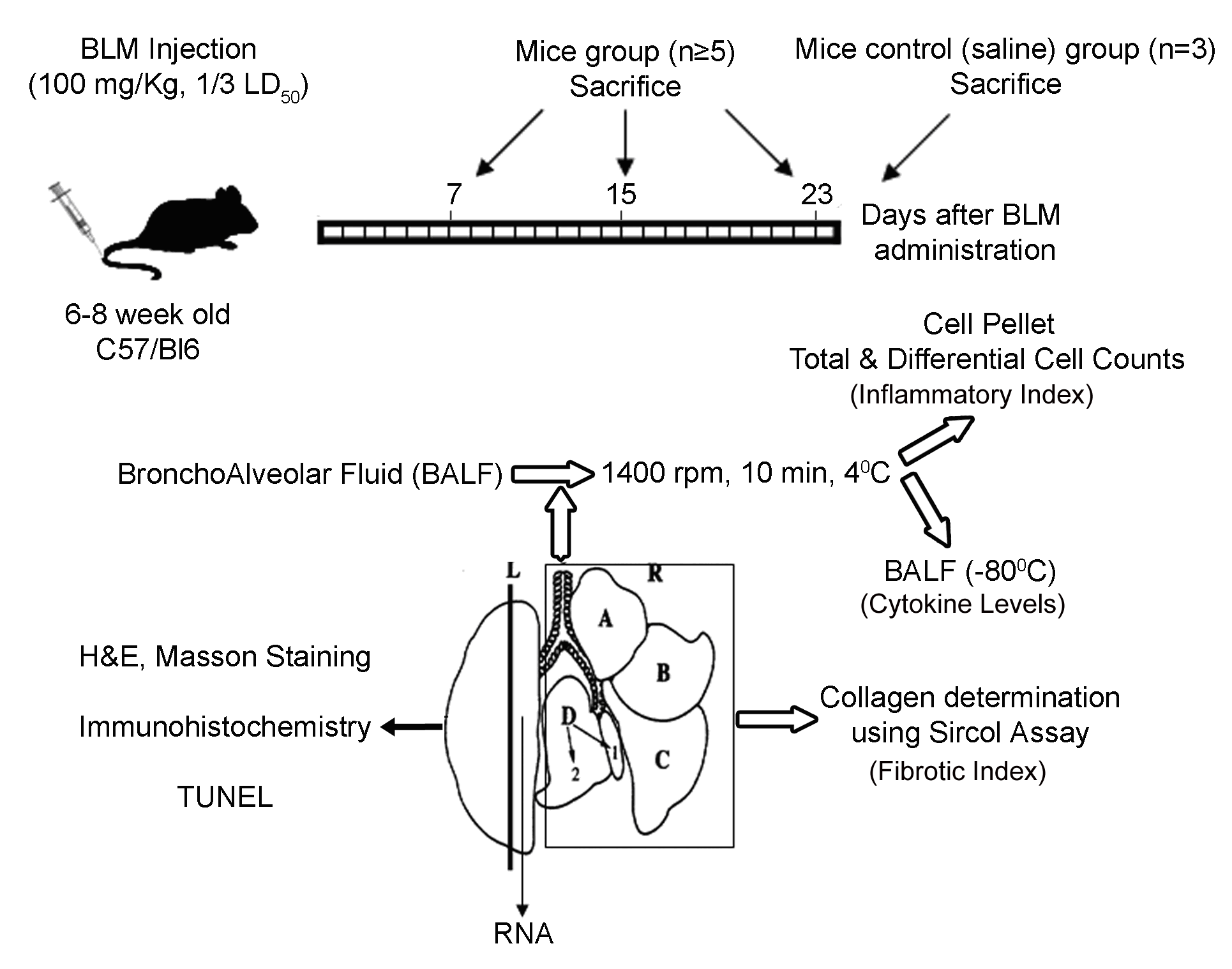


**Figure S1. Animal model of BLM-induced Pulmonary Fibrosis.**

In general, there are two types of animal models of diseases: natural models where the pathology appears without any intervention due to (spontaneous or engineered) genetic modifications and experimental models where the disease is induced through the administration of a pathogenic agent or through a pathogenic intervention (i.e. mechanical respiratory support). Because there is no natural model for Pulmonary Fibrosis, a variety of experimental models have been used, including administration of Bleomycin, Fluorescein Isothiocyanate, or particulate matter (such as silica and asbestos), as well as exposure to radiation [1]. Bleomycin (BLM) is an antibiotic agent with antitumor activity, commonly used for the treatment of several tumor types, such as germ-cell tumors, lymphomas, Kaposis’s sarcoma, cervical cancer and squamous cell carcinomas of head and neck [2]. BLM exerts its effects through the induction of free radicals and the formation of DNA double strand breaks, ultimately leading to cell death [3]. The application of BLM is featured by the occurrence of sometimes fatal side effects, due to the lack of the BLM-inactivating enzyme, Bleomycin hydrolase, in the lungs (and the skin). The pulmonary cytotoxicity, which frequently develops into fatal pulmonary fibrosis, led to the development of the BLM-induced animal model of PF. Original experimentation utilized parenteral routes of BLM administration such as intravenous (iv) to mimic the clinical application, but the intratracheal route of administration (IT) has become highly popular because PF can be induced with a relatively small amount of BLM, which moreover is (depending on the mouse strain) slightly less toxic [4]. In comparison, both routes of administration produce similar results in terms of collagen deposition and histopathological features (data not shown and [5]). On the contrary and most importantly, iv BLM administration produces subpleural lesions much like the human disease, while IT BLM administration produces peribronchiolar lesions (data not shown and [4]). Additionally, iv BLM administration is much less severe, thus allowing the detection of subtle differences in pathology, and exhibits slower kinetics (collagen production peaks at 23 days as compared to 14 days when utilizing the IT route) which allows for more thorough investigations of disease progression (data not shown and [4]). Overall, IT BLM administration is a much more acute model of Pulmonary Fibrosis thus resembling Acute Lung Injury (ALI) or Ventilator Induced Lung Injury (VILI) induced Pulmonary Fibrosis, while iv BLM administration is closer to the human BLM induced PF and IPF.

Therefore, PF was induced by a single tail vein injection of Bleomycin hydrogen chloride (100 mg/kg body weight; 1/3 LD50; Nippon Kayaku Co. Ltd., Tokyo) to 6- to 8-wk-old mice as previously described [6-8]. Of note, we never observed any lethality at the given dosage and mouse strain. Disease progression was monitored at 7, 15 and 23 days after Bleomycin injection, in parallel with control littermate mice injected similarly with saline (only at 23 d post injection).

At the appropriate endpoints mice were weighed and sacrificed; their lungs were lavaged three times with 1.0 ml aliquots of saline (BronchoAlveolar Lavage Fluid; BALF), followed by perfusion through the right ventricle of the heart with 10 ml of PBS. The lungs were then removed, weighed and dissected. BALF, after centrifugation at 1400 rpm for 10 min, were kept at -80^o^C for cytokine measurements, while cell pellets were used for total and differential cell counts with Trypan Blue and May-Giemsa staining respectively. The right lung was stored at -80^o^C for collagen determination with the Sircol collagen assay (Biocolor Ltd., Belfast, UK) essentially as previously reported [9]. The Sircol collagen assay is a well established direct quantitative method for the analysis of collagens (for a non-exhaustive list of publications please visit: www.biocolor.co.uk/manuals/Sircolreferences.pdf). It contains the reagent Sirius Red, an anionic dye with sulphonic acid side chain groups. These groups react specifically with the side chain groups of the basic amino acids present in collagen. The assay yields similar results with the hydroxyproline method, while it is much more reproducible (data not shown and www.biocolor.co.uk/manuals/Sircolmanual.pdf). In brief, right lungs were homogenized in PBS and centrifuged for 10 min at 2000 rpm. 50 μl of the supernatant was added to 1 ml of the Sircol dye. After shaking for 30 min the tubes were centrifuged for 5 min at 12000 rpm. Unbound dye solution was drained by turning the tubes upside down on a tissue, removing any remaining droplets from the top half of the tube with a tissue. After Alkali reagent addition (1 ml), the bound dye was released into the solution by vortexing. 100 μl of the solution was transferred to 96 well plate and the optical density was measured at 540_nm_ wavelength. Lung collagen content was calculated using a standard curve and expressed either as a % increase of saline treated lungs or as μg/Right Lung.

Finally, the left lung was divided sagittally; one half was snap-frozen in liquid N_2_ for RNA extraction while the other half was used for histopathology as previously reported [10].

1. Gharaee-Kermani M, Ullenbruch M, Phan SH (2005) Animal models of pulmonary fibrosis. Methods Mol Med 117: 251-259.

2. Sleijfer S (2001) Bleomycin-induced pneumonitis. Chest 120: 617-624.

3. Burger RM, Peisach J, Horwitz SB (1981) Activated bleomycin. A transient complex of drug, iron, and oxygen that degrades DNA. J Biol Chem 256: 11636-11644.

4. Lazo JS, Hoyt DG, Sebti SM, Pitt BR (1990) Bleomycin: a pharmacologic tool in the study of the pathogenesis of interstitial pulmonary fibrosis. Pharmacol Ther 47: 347-358.

5. Lindenschmidt RC, Tryka AF, Godfrey GA, Frome EL, Witschi H (1986) Intratracheal versus intravenous administration of bleomycin in mice: acute effects. Toxicol Appl Pharmacol 85: 69-77.

6. Hoyt DG, Lazo JS (1992) Murine strain differences in acute lung injury and activation of poly(ADP-ribose) polymerase by in vitro exposure of lung slices to bleomycin. Am J Respir Cell Mol Biol 7: 645-651.

7. Okudela K, Ito T, Mitsui H, Hayashi H, Udaka N, et al. (1999) The role of p53 in bleomycin-induced DNA damage in the lung. A comparative study with the small intestine. Am J Pathol 155: 1341-1351.

8. Piguet PF, Vesin C (1994) Pulmonary platelet trapping induced by bleomycin: correlation with fibrosis and involvement of the beta 2 integrins. Int J Exp Pathol 75: 321-328.

9. Phillips RJ, Burdick MD, Hong K, Lutz MA, Murray LA, et al. (2004) Circulating fibrocytes traffic to the lungs in response to CXCL12 and mediate fibrosis. J Clin Invest 114: 438-446.

10. Aidinis V, Carninci P, Armaka M, Witke W, Harokopos V, et al. (2005) Cytoskeletal rearrangements in synovial fibroblasts as a novel pathophysiological determinant of modeled rheumatoid arthritis. PLoS Genet 1: e48.
