# Supplementary figures and images for: Soluble TNF Mediates the Transition from Pulmonary Inflammation to Fibrosis
Source: PLoS One. 2006 Dec 27;1(1):e108. doi: 10.1371/journal.pone.0000108 (PMC1762410; doi:10.1371/journal.pone.0000108)

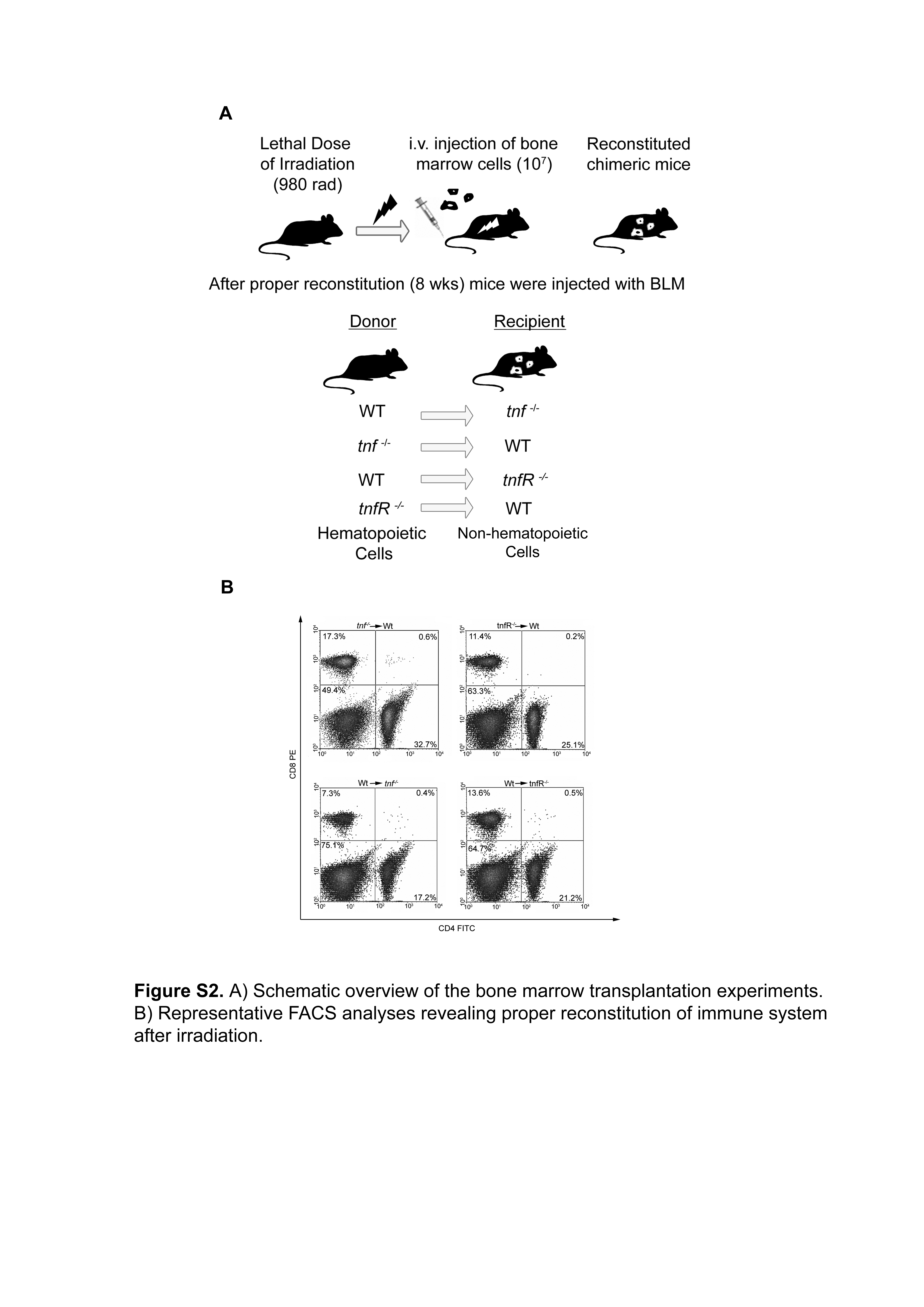

Supplement: Figure S2 — Schematic overview of bone marrow transplantation experiments. (1.62 MB TIF) [file pone.0000108.s002.tif]

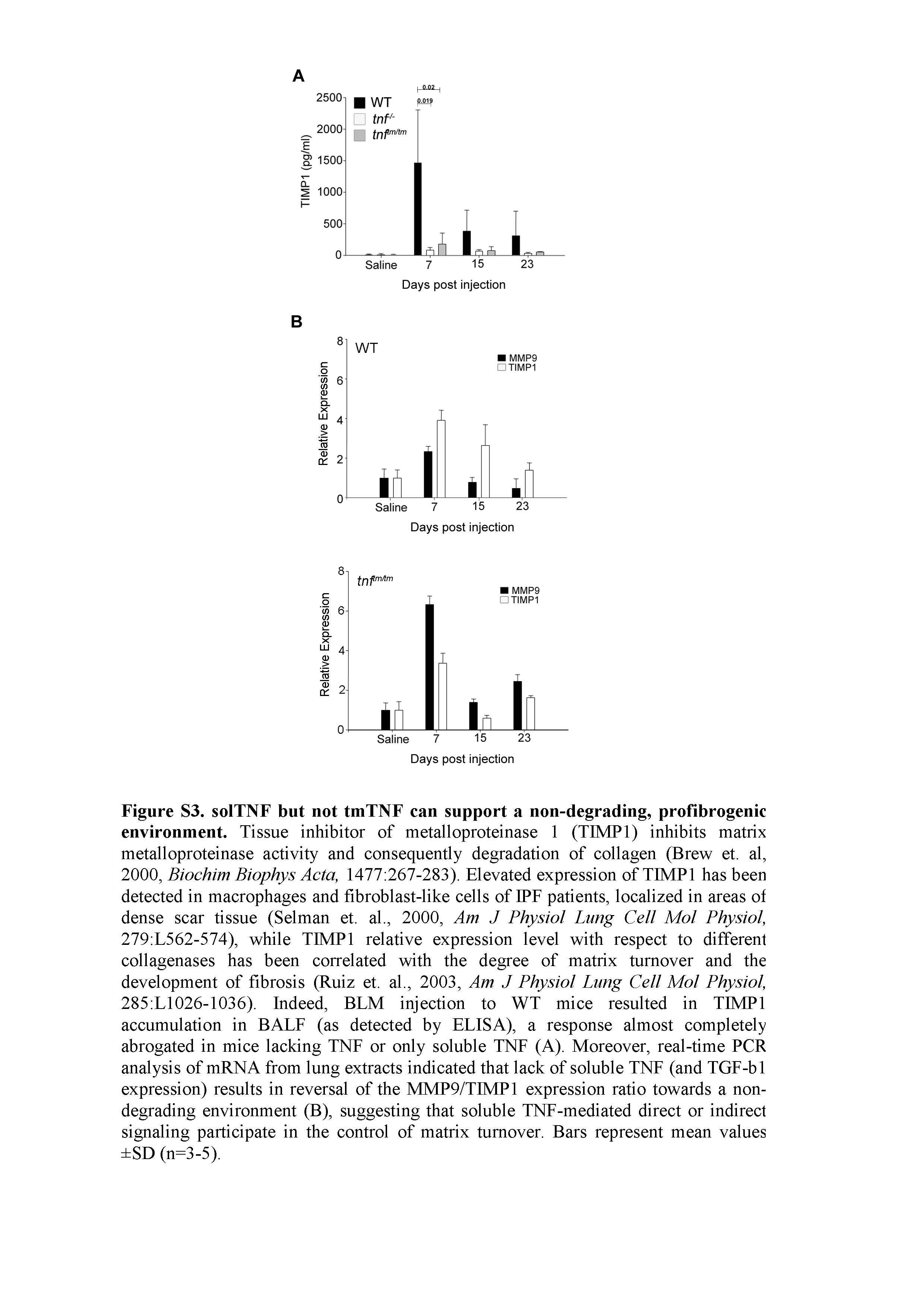

Supplement: Figure S3 — solTNF but not tmTNF can support a non-degrading, profibrogenic environment. (0.19 MB TIF) [file pone.0000108.s003.tif]
